# LSSP-PCR of *Trypanosoma cruzi*: how the single primer sequence affects the kDNA signature

**DOI:** 10.1186/1756-0500-6-174

**Published:** 2013-05-02

**Authors:** Marcela Segatto, Claudiney Melquíades Rodrigues, Carlos Renato Machado, Glória Regina Franco, Sérgio Danilo Junho Pena, Andréa Mara Macedo

**Affiliations:** 1 Departamento de Bioquímica e Imunologia, Instituto de Ciências Biológicas, Universidade Federal de Minas Gerais, Belo Horizonte, Minas Gerais, Brazil; 2 Laboratório de Genética Bioquímica, Departamento de Bioquímica e Imunologia, Instituto de Ciências Biológicas, Universidade Federal de Minas Gerais, Avenida Antônio Carlos, 6627 - Bloco K4, sala 245 - ICB/UFMG Bairro São Luiz, Belo Horizonte, Minas Gerais, CEP 31270-901, Brazil

**Keywords:** LSSP-PCR, *Trypanosoma cruzi*, kDNA signatures, Genetic diversity

## Abstract

**Background:**

Low-stringency single specific primer PCR (LSSP-PCR) is a highly sensitive and discriminating technique that has been extensively used to genetically characterize *Trypanosoma cruzi* populations in the presence of large amounts of host DNA. To ensure high sensitivity, in most *T. cruzi* studies, the variable regions of the naturally amplified kinetoplast DNA (kDNA) minicircles were targeted, and this method translated the intraspecific polymorphisms of these molecules into specific and reproducible kDNA signatures. Although the LSSP-PCR technique is reproducible under strict assay conditions, the complex banding pattern generated can be significantly altered by even a single-base change in the target DNA. Our survey of the literature identified eight different primers with similar, if not identical, names that have been used for kDNA amplification and LSSP-PCR of *T. cruzi*. Although different primer sequences were used in these studies, many of the authors cited the same reference report to justify their primer choice. We wondered whether these changes in the primer sequence could affect also the parasite LSSP-PCR profiles.

**Findings:**

To answer this question we compared the kDNA signatures obtained from three different and extensively studied *T*. *cruzi* populations with the eight primers found in the literature. Our results clearly demonstrate that even minimal modifications in the oligonucleotide sequences, especially in the 3′ or 5′ end, can significantly change the kDNA signature of a *T. cruzi* strain.

**Conclusions:**

These results highlight the necessity of careful preservation of primer nomenclature and sequence when reproducing an LSSP-PCR work to avoid confusion and allow comparison of results among different laboratories.

## Introduction

Chagas disease is caused by the protozoan *Trypanosoma cruzi* and has a variable clinical course ranging from symptomless infection to severe chronic disease with cardiovascular and/or gastrointestinal involvement. The factors influencing this clinical variability have not yet been elucidated, but both host and parasite genetic factors are likely important.

The biological, biochemical, and genetic diversity of *T. cruzi* strains have long been recognized, and over the years, numerous approaches have been used to characterize the parasites, such as multilocus enzyme electrophoresis (MLEE) [1,2], kinetoplast DNA restriction fragment length polymorphisms (kDNA RFLP) [3], random amplified polymorphic DNA (RAPD) [4-6], low-stringency single specific primer PCR (LSSP-PCR) [7], multilocus microsatellite typing (MLMT) [8-11], and many other methods such as PCR of the mini-exon and rDNA genes [12], [13].

The extensive efforts to comprehend the intraspecific genetic polymorphisms and population structure of *T. cruzi* are justified by the correlation between genetic variation and the biological properties of the parasite, including geographical distribution, host specificity, and the clinical outcome of infection. Additional understanding of the relationship between *T. cruzi* variation and clinical outcome will likely lead to a better understanding of the molecular epidemiology of Chagas disease [14]–[16].

In this context, LSSP-PCR targeting the sequence polymorphisms within the variable regions of *T. cruzi* kDNA [17]–[21] or the intergenic regions of the spliced-leader gene [22] allows direct profiling of the parasites present in the tissues of chronically infected patients [21], [23].

LSSP-PCR is an extremely simple, PCR-based technique that permits the detection of single or multiple mutations in gene-sized DNA fragments. Briefly, purified DNA fragments are subjected to PCR using high concentrations of a single specific oligonucleotide primer, large amounts of *Taq* DNA polymerase, and a very low annealing temperature. Under these conditions, the primer hybridizes specifically to its complementary region and nonspecifically to multiple sites within the DNA fragments in a sequence-dependent manner, producing a heterogeneous set of reaction products that constitutes a unique “gene signature profile” [24]. In fact, LSSP-PCR has been used in many organisms and fields of genetics and molecular medicine to obtain rapid, cheap and sensitive detection of mutations and sequence variations [25]–[33].

The first study to use LSSP-PCR on *T. cruzi* was performed by Vago and collaborators using a primer called S35 as a driver [21], [23]. This primer was originally designed to amplify minicircle variable region sequences of *T. cruzi*[34]. Many subsequent works cite these studies to justify the use of the chosen methodology, although the primer sequences published do not exactly match what was previously used. Although the LSSP-PCR technique is highly reproducible under strict conditions, the complex banding pattern obtained can be significantly altered by even a single-base change in the target DNA [24], which suggests that different primer sequences may also produce substantially different results.

Here, we surveyed the literature to catalogue the primer sequences used for *T. cruzi* kDNA analysis by LSSP-PCR and references cited by many research groups to assess the impact of the primer sequence on the parasite profiles. Our results clearly demonstrated that LSSP-PCR is a sensible and reproducible profiling technique, but minimal modifications in the oligonucleotide sequences, used in the second round of PCR, can significantly change the kDNA signature of *T. cruzi* strains.

## Findings

### Parasites and DNA extraction

Three *T. cruzi* populations were used: the CL Brener clone (*T. cruzi* VI), which was harvested from the CL strain isolated from a *Triatoma infestans* specimen; the Col1.7G2 clone (*T. cruzi* I), which was obtained from the Colombian strain and originally isolated from the blood of a chronic cardiac patient in Colombia; and the JG strain (*T. cruzi* II), a monoclonal population isolated from a chagasic patient with megaesophagus in Minas Gerais, Brazil.

*For T. cruzi* DNA extraction, the epimastigote forms of each parasite population were grown in liver infusion tryptose (LIT) medium containing 10% calf serum at 27–28°C. Once the culture contained 10^8^ epimastigote forms, the parasite cells were harvested, washed three times in sterile phosphate buffered saline and lysed in the presence of proteinase K overnight at 56°C. Standard DNA extraction was performed with phenol/chloroform as previously described [35].

### Low-stringency single specific primer polymerase chain reaction (LSSP-PCR)

The kDNA signatures were obtained using a two-step procedure. The first step consisted of the specific PCR amplification of fragments of approximately 330 bp from variable regions of *T. cruzi* kDNA minicircle molecules. This reaction was carried out in a final volume of 20 μl and contained 1.5 mM MgCl_2_, Green Go *Taq* Reaction Buffer pH 8.5 (Promega, Madison, Wisconsin, USA), 250 μM dNTPs, primers 121 or S35 (5′-AAATAATGTACGGGKGAGATGCATGA-3′) and 122 (5′-GGTTCGATTGGGGTTGGTGTAATATA-3′) at 1.0 μM, 1.0 U of Go *Taq* DNA Polymerase (Promega) and 1.0 ng of purified DNA template. Amplification was performed in a PT100 thermocycler (MJ Research) using an initial denaturation step at 94°C for 5 min followed by 35 amplification cycles of an annealing step at 60°C, extension at 72°C and denaturation at 94°C, each for 1 min. The final extension step was extended to 10 min. Five microliters of PCR products was visualized on a silver-stained 6% polyacrylamide gel as previously described [36].

The remaining 15 μL of the PCR reaction was then subjected to electrophoresis on an ethidium bromide-stained 1.5% agarose gel (1.0% agarose, 0.5% low melting point agarose). The kDNA amplicons were excised from the gel, diluted 10-fold in sterile Milli-Q water and submitted to a second step of low-stringency amplification using a single primer (LSSP-PCR) (Table 1). This second PCR was carried out in a final volume of 10 μl and contained 1.5 mM MgCl_2_, Colorless Go *Taq* Reaction Buffer pH 8.5 (Promega), dNTPs at 250 μM, primer at 4.5 mM, 1.6 U of Go *Taq* DNA Polymerase (Promega) and 1.0 μl of a solution containing the approximately 330 bp DNA fragments prepared as described above. Amplification was performed in a PT100 thermocycler (MJ Research) as follows: an initial denaturation step at 94°C for 5 min, followed by 40 amplification cycles of: an annealing step at 30°C, extension step at 72°C, and denaturation step at 94°C, each for1 min. The final extension step was extended to 10 min. LSSP-PCR products were also visualized on a silver-stained 6% polyacrylamide gel.

**Table 1:** **Sequence of primers designed to analyze three *T*. *cruzi *populations by LSSP-PCR**

**Primer code**	**Original name**	**Sequence (5′ → 3′)**	**Size (bp)**	**Reference**
		_**1 26**_		
**C**	*S35*	AAATAATGTACGGGKGAGATGCATGA	26	[20], [22], [34], [40]
**A**	*S35*	---ATAATGTACGGGKGAGATGC----	20	[42]
**B**	*S35*	AAATAATGTACGGG-GAGATGCATGA	25	[23], [43]–[45]
**D**	*S35*	AAATAATGTACGGG**G**GAGATGCATGA	26	[7], [21]
**E**	*S35G*	-----ATGTACGGG-GAGATGCATGA	20	[17], [35], [46]
**F**	S35G*	-----ATGTACGGG**G**GAGATGCATGA	21	[19]
**G**	*S35G*	AAATAATGTACGGG**G**GAGATG-----	21	[41], [47]
**H**	*S35G 5*	AAATAATGTACGGG**G**GAGAT------	20	Not published

*Indicates that this primer was originally described as fluorescein labeled but here the fluorescent marker was removed. All changes relative to primer S35 described by Sturm *et al*. (primer C) are highlighted.

### Data analysis

LSSP-PCR reactions were performed in triplicate, and only the consistent bands were taken into account to build a reproducible profile of each *T. cruzi* population. The multiband profiles obtained by LSSP-PCR of the *T. cruzi* populations were scored by eye, and each amplification band was numbered as present (1) or absent (0). These data were recorded on DNA-POP software [37], which calculates the proportion of shared bands among samples. Additionally, the distances among the profiles obtained with the different primers were calculated using the Nei and Li coefficient [38]. Phylogenetic trees were constructed based on genetic distance matrices obtained through UPGMA or primer sequences using the Treecon software program version 1.3b [39].

## Results

**Figure 1:**
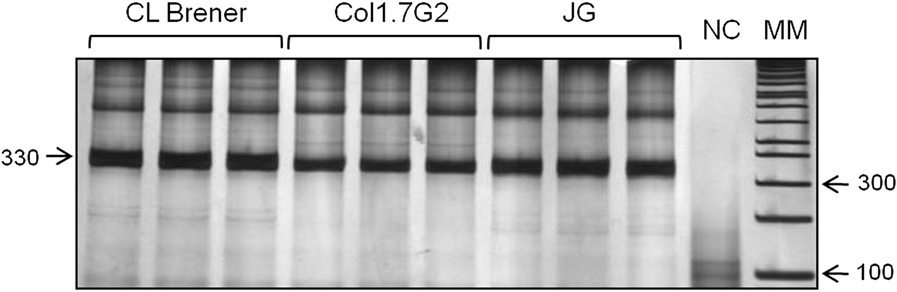
**Amplification of the hypervariable regions of kDNA minicircle molecules of three *T*. *cruzi *populations.** The PCR assays were performed in triplicate by three independent reactions for each *T. cruzi* population (CL Brener clone, Col1.7G2 clone or JG strain), as indicated in the figure. NC: negative control (without parasite DNA). MM: molecular size marker (1 Kb Plus DNA ladder - Invitrogen®).

The 330 bp band corresponding to the variable region of kDNA minicircle molecules was successfully amplified in all analyzed stocks (Figure 1). These amplicons were used as the templates for the second PCR reaction with different single primers to observe the influence of primer sequence on the kDNA signatures.

**Figure 2:**
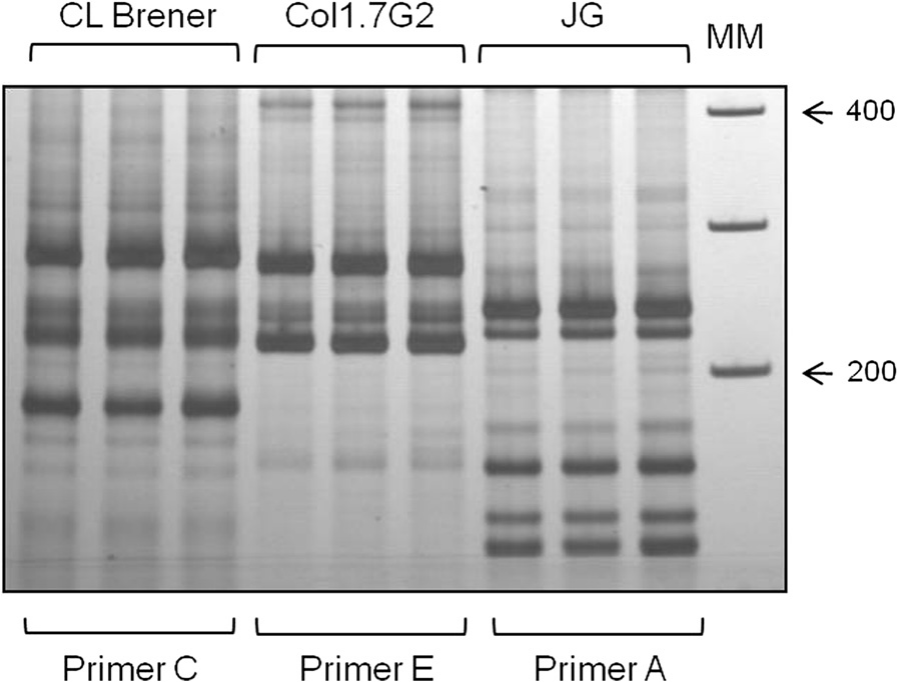
**Reproducibility of kDNA signatures obtained with different primers from LSSP-PCR.** The amplifications were performed in triplicate by three independent reactions for each *T. cruzi* population (CL Brener clone, Col1.7G2 clone or JG strain), each strain with a different primer, as indicated in the figure. MM: molecular size marker (1 Kb Plus DNA ladder - Invitrogen®).

**Figure 3:**
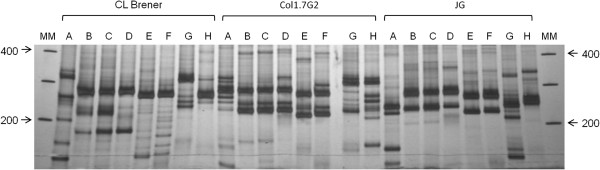
**Evaluation of the genetic variability among three *T. cruzi *populations by LSSP-PCR with different primers.** The primer used (A to H) is indicated below the profiles. MM: molecular size marker (1 Kb Plus DNA ladder - Invitrogen®).

The LSSP-PCR profiles were reproducible in the evaluated banding range of 100–400 bp (Figure 2) and showed high inter-strain genetic variability among the three *T. cruzi* analyzed stocks, with clear, distinct patterns for each strain that were independent of the primer used (Figure 3). However, the multiband profiles of each strain obtained with the eight different primers resulted mostly in different kDNA signatures, although there was minimal sequence variation observed for some primers (Figure 3).

**Figure 4:**
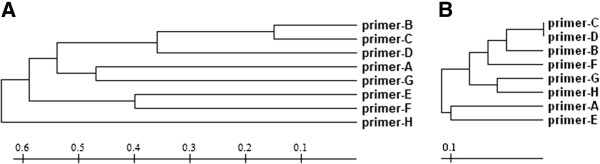
**LSSP-PCR trees.** (**A**) UPGMA tree generated from the distance of the LSSP-PCR profiles produced by each primer based on the sum of present and absent bands for the *T. cruzi* populations CL Brener, Col1.7G2 and JG. (**B**) Distance tree constructed from the primer sequences

We further investigated whether the LSSP-PCR profiles produced were more similar when the primer sequences were more similar. A phenotypic tree was constructed using the presence or absence of bands in the LSSP-PCR profiles as the distance matrix and the topology was compared with another tree constructed using the primer sequence data (Figure 4 and Table 1). As shown in Figure 4, there was not a general match between the trees’ topologies. For some of the primers (e.g., primers B, C and D) there was a correlation between the similarity of the LSSP-PCR profiles generated and the primer sequence similarities. However, this was not observed for other more heterogeneous primers, suggesting that the position of nucleotide substitutions or insertions/deletions may have a strong influence on the profiles. In this aspect, as expected, small differences in the 3′ region, such as the single deletion between primers G and H, caused dramatic changes in the LSSP-PCR profiles. However, unlike for conventional PCR, alterations in the 5′ region of the primers (e.g., primers D and F) also strongly affected the LSSP-PCR profiles (Table 1 and Figure 4). This was not observed for the primers B and D, where the deletion is located in a more central position of the primer sequences (15th nucleotide).

Finally, we used the median proportion of shared bands between the LSSP-PCR profiles derived from the different *T. cruzi* stocks to evaluate the ability of primers to distinguish different parasites. According to this analysis, the most discriminative primer was F generating an average of 24% shared bands among the three strains, whereas the less polymorphic was primer G with 48% of shared bands (Table 2).

**Table 2:** Average ratio of the proportion of shared bands among samples using the different primers

**Primer**	**Mean proportion of shared bands**
primer A	0.3909
primer B	0.4050
primer C	0.3882
primer D	0.3401
primer E	0.3945
primer F	0.2364
primer G	0.4873
primer H	0.3176
mean	0.3700

## Discussion

The application of LSSP-PCR to the characterization of the 330 bp variable portion of kDNA minicircle molecules produces complex banding patterns that allow identification of clones and strains from cultures or experimentally infected tissues with good discriminatory capacity [21]. Furthermore, the large genetic diversity in kDNA signatures obtained confirms the applicability of this method to genetic characterization studies on naturally infected vectors and humans [40], [41]. Differential tissue distribution of diverse clones of *T. cruzi* have been demonstrated in infected mice [35] and humans [7], [23]. LSSP-PCR is also useful to identify the differential distribution of *T. cruzi* populations associated with disease reactivation [18].

Despite being widely used for *T. cruzi* studies, LSSP-PCR reproducibility has been questioned due to its low-stringency nature. However, we have observed in our laboratory over our ten years of experience that LSSP-PCR patterns are highly reproducible even when the experiments are performed on separate days by different workers or when different thermocyclers and electrophoretic runs are used. To achieve this standardized amplification conditions both the enzyme and primer sources, and a good quality DNA at an adequate concentration must be consistently used [21]. Herein, we confirmed the high reproducibility of the technique by performing reactions at least three times on different days and obtaining highly stable profiles using different primers.

LSSP-PCR uses a single primer that hybridizes with high specificity to its complementary sequence incorporated into the amplicons during the first round of PCR, and also with low specificity but in a sequence-dependent manner to multiple sites within the amplified fragment during the second round. Thus the reaction yields a large number of products that can be resolved by electrophoresis to give rise to a multiband DNA fragment signature that reflects the DNA template sequence [24]. Changes as small as a single base mutation could drastically alter the multiband pattern, producing new signatures that are diagnostic of the specific alterations [21], [24]. In this context, we asked whether the primer sequence might also influence the kDNA complex band in pattern.

To that end, we evaluated eight primer sequences previously used in the literature with similar names or citations. Our results demonstrated that sets of primers with related sequences, but differing from one another by 1 to 7 bases, resulted in different kDNA signatures for the same strain. In fact, alterations in primer sequence as small as a unique base mutation affected the kDNA multiband patterns, especially changes in the 3′or 5′ regions.

On the other hand, when we analyzed the profiles obtained with the same primer for the three evaluated strains, we saw that they were completely different from one another despite of the primer used. This is extremely important since the main goal of LSSP-PCR is to detect genetic polymorphisms in *T. cruzi* isolates belonging to distinct populations such as in different distant endemic areas or outbreaks or different clones within the same population with different tropisms, for example.

In conclusion we demonstrate here the importance of primer sequence when performing LSSP-PCR, at least for *T. cruzi* kDNA. This is especially relevant because different researchers frequently reproduce published techniques, but if the primer sequences are not faithful, comparisons of LSSP-PCR results among laboratories are not feasible, contributing to the false idea that LSSP-PCR is a technique poorly reproducible. Additionally, cases where oligonucleotide sequences are intentionally changed should be followed also by changes in the primers’ names. This would be highly useful for all laboratories working on kDNA signatures, while avoiding confusion and improving the comparison of LSSP-PCR patterns among laboratories.

## Competing interests

The authors declare that they have no competing interests.

## Authors’ contributions

MS processed the samples and experiments, analyzed data, reviewed the literature and wrote the manuscript. CMR processed the samples and experiments, analyzed data and reviewed the manuscript. CRM, GRF and SDJP analyzed data and reviewed the manuscript. AMM conceived of the study, and participated in its design and coordination and reviewed the manuscript. All authors read and approved the final manuscript.
